# The Association between the Body Mass Index, Chronic Obstructive Pulmonary Disease and SUV of the Non-Tumorous Lung in the Pretreatment [^18^F]FDG-PET/CT of Patients with Lung Cancer

**DOI:** 10.3390/diagnostics14111139

**Published:** 2024-05-30

**Authors:** Lukas Wehlte, Julia Walter, Lea Daisenberger, Felix Kuhnle, Maria Ingenerf, Christine Schmid-Tannwald, Matthias Brendel, Diego Kauffmann-Guerrero, Lucie Heinzerling, Amanda Tufman, Thomas Pfluger, Friederike Völter

**Affiliations:** 1 Department of Medicine V, LMU University Hospital, 80336 Munich, Germany; 2 German Center for Lung Research (DZL CPC-M), 81377 Munich, Germany; 3 Department of Dermatology and Allergy, LMU University Hospital, 80336 Munich, Germany; 4 Department of Radiology, LMU University Hospital, 80336 Munich, Germany; 5 Department of Nuclear Medicine, LMU University Hospital, 80336 Munich, Germany; 6 Munich Cluster for Systems Neurology (SyNergy), 81377 Munich, Germany; 7 German Center for Neurodegenerative Diseases (DZNE), 81377 Munich, Germany; 8 Department of Dermatology, University Hospital Erlangen, Comprehensive Cancer Center Erlangen—European Metropolitan Region Nürnberg, CCC Alliance WERA, 91054 Erlangen, Germany

**Keywords:** lung cancer, NSCLC, SCLC, PET, PET/CT, SUV, immunotherapy, BMI, COPD, scanner

## Abstract

*Background*: A debate persists on the prognostic value of the pre-therapeutic standardized uptake value (SUV) of non-tumorous lung tissue for the risk assessment of therapy-related pneumonitis, with most studies lacking significant correlation. However, the influence of patient comorbidities on the pre-therapeutic lung SUV has not yet been systematically evaluated. Thus, we aimed to elucidate the association between comorbidities, biological variables and lung SUVs in pre-therapeutic [^18^F]FDG-PET/CT. *Methods*: In this retrospective study, the pre-therapeutic SUV in [^18^F]FDG-PET/CT was measured in non-tumorous areas of both lobes of the lung. SUV_MEAN_, SUV_MAX_ and SUV_95_ were compared to a multitude of patient characteristics and comorbidities with Spearman’s correlation analysis, followed by a Bonferroni correction and multilinear regression. *Results*: In total, 240 patients with lung cancer were analyzed. An elevated BMI was significantly associated with increased SUV_MAX_ (β = 0.037, *p* < 0.001), SUV_MEAN_ (β = 0.017, *p* < 0.001) and SUV_95_ (β = 0.028, *p* < 0.001). Patients with chronic obstructive pulmonary disease (COPD) showed a significantly decreased SUV_MAX_ (β = −0.156, *p* = 0.001), SUV_MEAN_ (β = −0.107, *p* < 0.001) and SUV_95_ (β = −0.134, *p* < 0.001). Multiple other comorbidities did not show a significant correlation with the SUV of the non-tumorous lung. *Conclusions*: Failure to consider the influence of BMI and COPD on the pre-therapeutic SUV measurements may lead to an erroneous interpretation of the pre-therapeutic SUV and subsequent treatment decisions in patients with lung cancer.

## 1. Introduction

Lung cancer is one of the most common and deadly malignancies worldwide. Its global annual incidence is still increasing [1,2]. Therapy with immune checkpoint inhibitors (ICI) dramatically improved survival in patients with advanced-stage disease at first diagnosis [1,3,4,5,6,7]. However, severe immunotherapy-related adverse effects (irAEs) can occur during immune checkpoint inhibitor therapy and up to now are unpredictable. Approximately one out of every ten patients develops immune checkpoint inhibitor-associated pneumonitis (CIP) [8,9]. CIP has a major effect on the lung function, quality of life and overall survival of patients [9]. Higher-stage CIP can be life-threatening and often leads to a permanent discontinuation of immune checkpoint inhibitor therapy [10,11]. Hence, it is imperative to discern which patients are predisposed to developing CIP and which are not.

Recently, several studies investigated the pre-therapeutic standardized uptake value (SUV) of non-tumorous lung tissue in [^18^F]-fluorodeoxyglucose positron emission tomography/computed tomography (FDG-PET/CT) as a potential predictive biomarker for the risk of developing radiation therapy-associated and immune checkpoint inhibitor associated pneumonitis [12,13,14,15,16,17,18,19,20,21]. In individuals undergoing radiation therapy, a significant partial correlation exists between an increased pre-radiotherapeutic PET-measured glucose uptake and an elevated risk of radiation-induced pneumonitis [12,15]. However, most studies focusing on the predictability of *immunotherapy-associated* pneumonitis failed to establish a significant correlation between pre-therapeutic FDG uptake and the risk of therapy-related pneumonitis [16,17,18,19,20,21]. In the studies referenced, the impact of patients’ comorbidities on the SUV was not incorporated into the analyses. However, patients with lung cancer often show smoking-related comorbidities like chronic obstructive pulmonary disease (COPD) [22,23]. In these patients, an increased partial volume of trapped air could potentially lead to a lower SUV. On the contrary, in patients with obesity, a higher amount of mediastinal and abdominal fat could potentially lead to a higher lung density due to a functional restriction [24] and therefore to an elevated SUV of the lung tissue. There are many more biological variables and comorbidities potentially influencing the SUV of the lung and complicating the interpretation (e.g., cardiac congestion, pleural effusion, varying PET/CT scanner).

To ensure a correct interpretation of the SUV in non-tumorous lung tissue as a potential predictor for adverse events, understanding the impact of patient comorbidities on the pre-therapeutic SUV is imperative. To our knowledge, in patients with lung cancer, the impact of comorbidities and other clinical variables on the SUV of the non-tumorous lung has not yet been systematically evaluated. Thus, we aimed to investigate the influence of comorbidities and biological variables on the pre-therapeutic SUV of non-tumorous lung tissue in FDG-PET/CT, with the ultimate goal of improving pre-therapeutic assessment and therapy planning.

## 2. Materials and Methods

### 2.1. Patient Population

All patients with lung cancer who were treated with an immune checkpoint inhibitor therapy at the Department of Medicine V at LMU Klinikum between 2014 and 2022 and who were staged with FDG-PET/CT before the start of ICI therapy/combination therapy of ICI with chemotherapy and/or radiation therapy were included. Patients who received their FDG-PET/CT after or more than one year before the start of immunotherapy were excluded. This study was conducted in accordance with the Declaration of Helsinki and approved by the ethics committee der LMU München (Project No. 474-16 UE).

### 2.2. FDG-PET/CT Imaging

Before injection of the radiotracer, patients fasted for 4–6 h. At the time of injection, blood glucose levels were below 150 mg/dL. The scans were acquired in mid-breath-hold. The SUV was then calculated using the following standard calculation [21].

SUV calculation:

(1) $$SUV=\frac{Sphere\;activity\left[\frac{Bq}{ml}\right]*body\;weight\left(kg\right)}{injected\;dose}$$

### 2.3. Image Analysis

**Figure 1:**
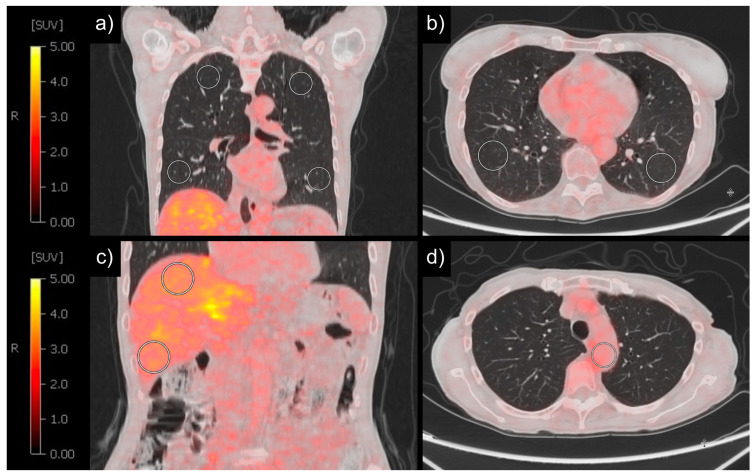
Example of PET/CT analysis with spheres. SUV measurement with spheres with 30 mm diameter in lung in coronal view (**a**), lung in axial view (**b**), and liver in coronal view (**c**). SUV measurement with one sphere and diameter depending on aorta size in blood localized in aorta in axial view (**d**).

FDG-PET/CT images were evaluated using Visage^®^7, Visage Imaging, Inc. (San Diego, CA, USA; version 7.1.18). Four three-dimensional spheres with a diameter of 30 mm were placed into lung parenchyma, ensuring placement in regions not infiltrated by lung tumor. One sphere was placed in each of the upper and lower lobes of both the right and left lungs, respectively (Figure 1). Spheres were excluded when it was not possible to place them exclusively in non-tumorous lung tissue (e.g., due to tumor infiltration, metastasis, or lobectomy). For normalization, one sphere was placed in the lumen of the aorta and two additional spheres were placed in liver tissue. The SUV (SUV_MEAN_), maximum SUV (SUV_MAX_), and the standard deviation (SD) of the SUV (SUV_SD_) were measured for each sphere. Mean values were measured for the SUV_MEAN_ and SUV_MAX_ of all four spheres. Additionally, mean values were computed for the two upper spheres, the two lower spheres and the two left or right spheres depending on tumor side (TFL). Furthermore, for normalization ratios were calculated between the SUV_MEAN_ values obtained from all four spheres and those from the two liver spheres, as well as the sphere in the mediastinal blood pool. Lastly, SUV_95_ was measured for all four spheres. SUV_95_ describes the 95th percentile of SUV_MAX_ and was calculated with the following formula:

SUV_95_ measurement:

(2) $${SUV}_{95}={SUV}_{MEAN}+(qnorm(0.95)*{SUV}_{SD})$$

### 2.4. Patient Characteristics

The following variables with a potential impact on the SUV were collected: sex, age, BMI, tumor stage, tumor side, smoking status, pack years, previous lung operation, previous lung radiation, COPD, diabetes mellitus type II, asthma, coronary heart disease (CHD), pericardial effusion (PCE), pleural effusion (PE), pneumonia (no more than three months before PET) and blood hemoglobin (Hb) level. A semi-automatic tool was built to analyze the text of electronic patient files using keywords for those characteristics. Possible spelling errors were considered. When keywords were found, the text was read manually. All collected data were anonymized and stored in a Microsoft Excel database (Redmond, WA, USA; version 2108).

### 2.5. Statistical Analyses

Categorical patient characteristics and clinical variables were summarized using absolute and relative frequencies. Following the Lilliefors normality test, the mean and standard deviation were calculated for Gaussian-distributed numerical variables, while the median and interquartile range (IQR) were computed for non-Gaussian-distributed numerical variables. Kruskal–Wallis test was used to test if mean SUV_MEAN_, mean SUV_MAX_ and SUV_95_ were significantly different when using different PET/CT scanners. To determine the influence of different variables on SUV, Mann–Whitney U test was used for binary variables, and Spearman’s correlation analysis was used for numerical variables, with each of them being followed by a Bonferroni correction of the significance level for multiple testing. Additionally, a multilinear regression was performed, and the resulting beta coefficients were visualized in a forest plot. The association between biological variables and comorbidities and the SUV was evaluated for all patients as well as for the largest subgroup of patients that were scanned on the same PET/CT scanner. Boxplots and a scatterplot were used for the visualization of the test results. All statistical analyses and plots were conducted using R-Studio (Boston, MA, USA; version 2023.09.1+494) and R (Vienna, Austria; version 4.3.2).

## 3. Results

### 3.1. Patient Characteristics

A total of 293 patients underwent ICI therapy or combination therapy of ICI therapy with chemotherapy (*n* = 164) and/or radiation therapy (*n* = 63) as a first-line or subsequent therapy during the observation period. In total, 53 patients were excluded because the FDG-PET/CT was acquired after the start of ICI therapy or more than one year before ICI therapy commenced. The data of the remaining 240 patients are displayed in Table 1. PET/CT data from fifteen different locations were used for this analysis. In 137/293 patients, the pre-therapeutic FDG-PET/CT was acquired using the same imaging PET/CT scanner (GE Healthcare Discovery PET/CT 690 at LMU Klinikum).

### 3.2. Association of Biological Variables and Semiquantitative PET Parameters

Mean SUV_MEAN_ of the lung varied between 0.16 g/mL and 1.37 g/mL. Mean SUV_MAX_ of the lung varied between 0.31 g/mL and 2.63 g/mL; mean SUV_95_ varied between 0.16 g/mL and 1.94 g/mL. Prior to the correlation analysis, the congruence of SUV_MEAN_, SUV_MAX_ and SUV_95_ obtained from various PET/CT scanners was tested with the Kruskal–Wallis test (Table A1). While mean SUV_MEAN_ and SUV_95_ was comparable across all PET/CT scanners (mean SUV_MEAN_: *p* = 0.193–0.356 and SUV_95_: *p* = 0.075–0.591), there were significant differences regarding the mean SUV_MAX_ (*p* = 0.007–0.160) and lung/blood ratio (*p* = 0.004–0.017). Thus, all analyses were not only carried out using the entire cohort of 240 patients but also verified within the subset consisting of 137 patients who underwent scanning with the identical PET/CT scanner.

**Figure 2:**
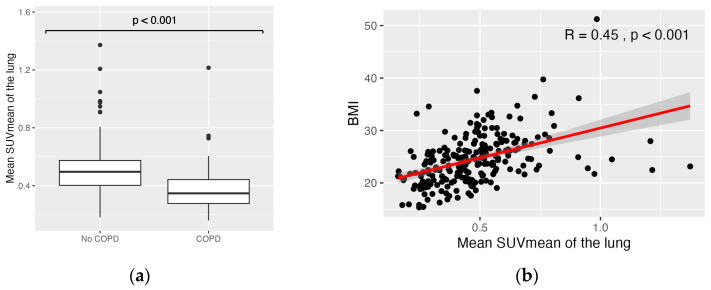
SUV_MEAN_ of patients with and without COPD was significantly different (**a**); moderate correlation of SUV_MEAN_ and BMI (**b**). COPD = chronic obstructive pulmonary disease, BMI = body mass index, SUV = standardized uptake value. The red line corresponds to the regression line.

After the Bonferroni correction, BMI, smoking, pack years and COPD showed a significant correlation with the SUV (Table 2, Figure 2). The most robust correlation with tracer uptake was shown in the correlation analysis of the SUV of the whole lung and BMI (R = 0.32–0.58, *p* < 0.001). Furthermore, there were significantly decreased SUVs in patients with COPD (mean SUV_MEAN_: 0.38 g/mL vs. 0.51 g/mL, *p* < 0.001; mean SUV_MAX_: 0.77 g/mL vs. 0.97 g/mL, *p* < 0.001; mean SUV_95_: 0.54 g/mL vs. 0.71 g/mL, *p* < 0.001). The significant correlation of BMI and COPD with SUV_MEAN_ and SUV_MAX_ was confirmed in the subcohort of 137 patients who underwent scanning with the identical PET/CT scanner (Table A2). There was a variety of clinical parameters showing no significant correlation with the evaluated PET parameters: sex, age, tumor stage, lung operations, thorax radiotherapy, pleural effusion, pericardial effusion, diabetes mellitus type II, coronary heart disease and Hb did not show a significant effect on the SUV of non-tumorous lung-tissue. Normalization of the lung SUV to the SUV of the liver or the SUV of the mediastinal blood pool yielded equivalent results. 

**Figure 3:**
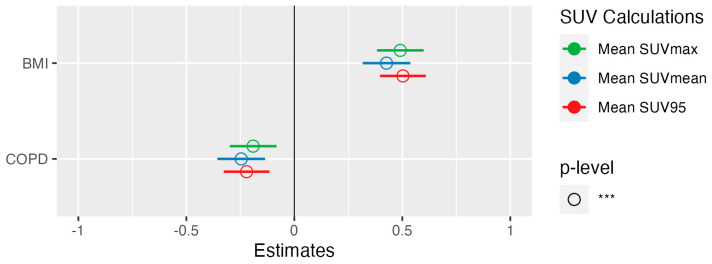
Forest plot of multilinear regression with SUV as response, and biological variables as independent variables. COPD = chronic obstructive pulmonary disease, BMI = body mass index, SUV = standardized uptake value, *** = *p* < 0.05.

**Figure 4:**
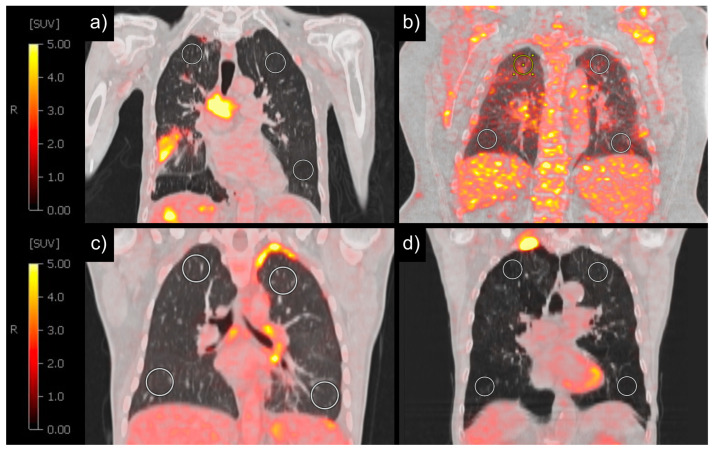
SUV measurements of the non-tumorous lung in four patients with a low BMI (15.3 kg/m^2^), resulting in a correspondingly reduced SUV_MEAN_ (0.25 g/mL) (**a**); with a high BMI (51.2 kg/m^2^), resulting in a correspondingly higher SUV_MEAN_ (0.99 g/mL) (**b**); without COPD (SUV_MEAN_ = 0.47 g/mL) (**c**); and with COPD (SUV_MEAN_ = 0.17 g/mL) (**d**). BMI = body mass index, SUV = standardized uptake value, COPD = chronic obstructive pulmonary disease.

Multilinear regression was used to confirm the association between the previously significant clinical parameters in Table 2 and the PET parameters SUV_MEAN_, SUV_MAX_ and SUV_95_. Only one of the smoking-related parameters (pack years and COPD) was used as an independent variable for multilinear regression, leaving BMI and COPD as independent variables. In multilinear regression, patients with increased BMI showed significantly increased mean SUV_MAX_ (β = 0.037, *p* < 0.001), mean SUV_MEAN_ (β = 0.017, *p* < 0.001) and SUV_95_ (β = 0.028, *p* < 0.001) (Figure 3, Figure 4a,b). Patients with COPD, on the other hand, showed a significantly decreased mean SUV_MAX_ (β = −0.156, *p* = 0.001), mean SUV_MEAN_ (β = −0.107, *p* < 0.001) and SUV_95_ (β = −0.134, *p* < 0.001) compared to the patients without COPD (Figure 3, Figure 4c,d). These results could be confirmed in the subcohort of 137 patients who underwent scanning with the identical PET/CT scanner (S3).

## 4. Discussion

Predictors for severe immune-related adverse events (irAEs) induced by immune checkpoint inhibitor therapy are desperately needed. Immune-related pneumonitis is the ICI-induced adverse event causing the most fatalities [10,11,25,26]. Thus, radiologic assessments from routine care carry a high potential to guide monitoring and treatment decisions if correlations with subsequent toxicity could be identified. The standardized uptake value (SUV) in FDG-PET/CT is gaining increasing interest, which is reflected by the wealth of research around the SUV as a predictor of treatment response and therapy-related adverse events (AEs). While most research has focused on the assessment of the SUV in tumor tissue as a prognostic marker, only few studies focused on the FDG uptake in healthy organs to identify patients with cancer at risk of AEs. Particularly in the context of immune-related adverse events (irAEs) associated with immune checkpoint inhibitor therapy, the SUV of non-tumorous tissue could provide a predictive advantage. The paramount aim is to identify, prior to treatment, patients with cancer prone to immunotherapy-associated adverse events through pre-therapeutic FDG-PET/CT. This would facilitate closer monitoring of susceptible patients, prompt therapy adjustments, and potentially enable a timelier integration of (inhalative) corticosteroids or other immune modulators into treatment protocols. Previous studies showed that an increased tracer uptake *during* immunotherapy reflected the presence of active immunotherapy related or radiation-therapy related adverse events [16,27,28]. Nevertheless, the majority of studies assessing the SUV *before* immunotherapy as a predictive biomarker for immune-related adverse events (irAEs) have yielded inconclusive results, failing to demonstrate a significant correlation between the pre-therapeutic SUV and the occurrence of ICI-induced irAEs [16,17,18,19]. Focusing on checkpoint inhibitor-related pneumonitis (CIP), only one study successfully predicted the probability of irAEs during therapy with pre-therapeutic PET, considering the CT and the combined PET/CT information in a radiomics score [21]. The SUV of the lung can be heavily influenced by confounding factors, including sex, body mass index (BMI) and diabetes [29,30,31]. Furthermore, comorbidities with an impact on tissue density such as lung emphysema could have a significant impact on the lung SUV and should therefore be considered when evaluating the pre-therapeutic FDG uptake in non-tumorous lung tissue. Notwithstanding the numerous studies examining the prognostic utility of SUV for the prediction of CIP in patients with lung cancer, a systematic exploration of the impact of comorbidities on the non-tumorous lung SUV within this patient cohort remains outstanding. Given this background, we aimed to elucidate the effect of biological variables and common comorbidities of patients with lung cancer on the pre-therapeutic SUV of the non-tumorous lung.

In our study, a higher BMI was consistently strongly associated with a higher SUV_MEAN_, SUV_MAX_ and SUV_95_. When reviewing the literature, little information can be found on the influence of BMI on lung SUV [32,33]. A previous study investigating the effect of blood glucose levels on the SUV of healthy organs in over 5600 patients showed that an elevated BMI was significantly associated with an increased SUV of the lung [33]. Similarly, a small-scale study comprising 18 subjects demonstrated a significant association between a higher BMI and an increased SUV of the lung, implying the necessity for validation in a larger sample size [32]. The increase in mediastinal and intra-abdominal fat accompanying higher BMI levels likely contributes to elevated intra-thoracic pressure, a lower total lung capacity and an increased lung tissue density [34]. As the SUV is calculated based on measured radioactivity within a defined voxel, greater lung tissue density, with less air, results in higher SUV values. Conversely, reduced lung tissue density, with more air, leads to decreased SUV values. Additionally, a systemic chronic inflammatory response often associated with higher BMI levels might have contributed to the correlation between the BMI and the lung SUV [35,36,37]: an elevated BMI has been linked to increased levels of pro-inflammatory cytokines and adipokines, which could potentially enhance metabolic activity and inflammation within lung tissue, thereby influencing SUV measurements [35,36,37]. However, we also investigated a potential association of diabetes and the pre-therapeutic lung SUV, which is another comorbidity with a known influence on metabolic activity and is associated with chronic inflammatory activity. Our analysis did not reveal a significant correlation between diabetes status and lung SUV. Thus, although chronic inflammatory processes may indeed play a role in modifying metabolic activity within lung tissue, it is conceivable that factors influencing lung tissue density could exert a predominant influence on SUV measurements in this context. While a large study of over 5000 patients found that obesity paradoxically reduces the risk of pulmonary and other complications after minimally invasive thoracic surgery [38], in the context of checkpoint inhibitor therapy, multiple studies showed that an elevated BMI was linked to the occurrence of CIP [39,40,41]. In light of the substantial impact of the BMI on the lung SUV, we draw the conclusion that when investigating the relationship between the pre-therapeutic SUV and the risk of CIP, it is imperative to recognize that an elevated pre-therapeutic SUV may not exclusively reflect heightened inflammatory activity. Instead, pre-therapeutic SUVs are also influenced by increased tissue density and potentially altered metabolic activity associated with a higher BMI. Moreover, our findings highlight the potential to enhance the precision of discriminating between lung lesions through the inclusion of the BMI in the analysis. An SUV threshold of 2.5 has been the gold standard for discriminating between malignant and benign lung lesions [42,43]. Prior research suggests that the correlation between the tumor SUV of lung lesions and prognosis is stronger in normal weight individuals compared to their obese counterparts [44,45]. For patients with breast cancer, it was already shown that risk stratification using a tumor SUV could be ameliorated when using the BMI as a covariable independent of tumor subtype and clinical stage [46]. Consequently, there might be merit in considering the incorporation of patients’ BMI into the risk assessment framework for lung lesions.

Despite the acknowledged increase in inflammatory activity of the lungs in patients with COPD [47,48,49], our study consistently revealed a significantly lower SUV in the non-tumorous lung of patients with chronic obstructive pulmonary disease. COPD and smoking are linked to lung emphysema, manifesting as decreased tissue density [50] and potentially as a decreased density of glucose uptake. Our results therefore suggest a predominant effect of the decreased tissue density over the increased inflammatory activity of the lungs in patients with COPD. Previous studies showed an increased risk for CIP in patients with COPD and lung emphysema [51,52]. Considering that COPD is associated with a decreased SUV, a negative association between the SUV and the occurrence of pneumonitis would be expected. Based on our findings, we advocate for the distinct examination of SUV measurements within patient subgroups stratified by a COPD status and for the inclusion of COPD as a covariate in the analytical models.

It is recognized that for optimal patient care and comparability of PET scans, ideally, all imaging should be conducted using the same PET scanner [30]. However, upon comparing the standardized uptake values obtained from multiple PET/CT scanners, we observed that SUV_MEAN_ and SUV_95_ were consistent across the diverse array of PET/CT scanners analyzed in this study. Nevertheless, disparities were evident in SUV_MAX_, probably resulting from varying reconstruction algorithms and post-processing methods, e.g., the application of different smoothing algorithms, potentially leading to an underestimation of SUV_MAX_ [30,53]. To accommodate for possible confounders, we performed all statistical tests using the data of all included patients and with a subgroup that was scanned on the most frequently used PET/CT scanner (*n* = 137). These subgroup re-tests confirmed the results of the whole patient group, showing a highly significant impact of COPD and the BMI on lung SUV. In summary, our results suggest a sufficient comparability of SUV_MEAN_ and SUV_95_ of imaging studies acquired with varying PET scanners. This finding could be useful in everyday clinical practice when patients come in for staging evaluations with FDG-PET/CT scans and have had PET imaging completed elsewhere before. In these patients, the 95th percentile of SUV_MAX_ (SUV_95_) could be used instead of SUV_MAX_ to assess disease progression. However, the comparability of the SUV_95_ of benign and malignant structures in FDG-PET/CT has to be confirmed in future studies.

The following limitations have to be considered when interpreting this study: First, the study cohort exclusively included patients receiving FDG-PET/CT before checkpoint inhibitor therapy. This might have led to a selection bias. Furthermore, this study is limited by its retrospective design. As the medical documentation was not primarily intended for this research, clinical information about patient comorbidities may have been incomplete.

## 5. Conclusions

Our study highlights a significant link between COPD, the BMI, and the pre-therapeutic lung SUV. In obese patients, a physiologically higher SUV in non-tumorous lung areas should be factored into the assessment of therapy-related pneumonitis risk. Moreover, in patients with a higher BMI, malignant lung lesions may show reduced contrast to surrounding non-tumorous lung tissue, potentially complicating diagnosis. Conversely, in patients with COPD, a lower SUV in non-tumorous lung regions may obscure inflammatory activity. In conclusion, acknowledging the influence of obesity and COPD is crucial for an accurate SUV interpretation. Future research should focus on tailored risk management of pre-therapeutic inflammatory activity and the assessment of malignancy risk of lung nodules in patients with obesity and COPD.

## Figures and Tables

**Table 1:** Patient, tumor, PET/CT and treatment characteristics of the included 240 patients.

Variable	Mean/Median	+/− SD/IQR
Physical characteristics		
Age [y]	68.0	14.25
BMI [kg/m≤]	24.49	4.67
Smoking history		
Pack years [y]	34.39	26.95
Blood count		
Hb [g/dL]	12.0	3.18
Variable	No.	%
Physical characteristics		
Biological sex		
Female	91	37.9
Male	149	62.1
Tumor		
Staging		
I	2	1.5
II	46	33.6
III	88	64.2
IV	1	0.7
Histology		
NSCLC adenocarcinoma	137	57.1
NSCLC squamous cell carcinoma	65	27.1
SCLC neuroendocrine carcinoma	24	10
Other lung cancer histology ^1^	14	5.8
Clinical history		
Thorax radiation before FDG-PET/CT	63	26.3
Lung operation	23	9.6
Smoking history		
Nicotine consumption	198	82.5
in female patients	69	75.8
in male patients	129	86.6
Comorbidities		
COPD	57	23.8
Pericardial effusion	15	6.3
Pleural effusion	57	23.8
Diabetes mellitus type II	36	15.1
Coronary heart disease	34	14.2
PET/CT Scanner		
GE Discovery 690	137	57.1
Siemens Biograph 20	42	17.5
Siemens Biograph 40	19	7.9
Siemens Biograph 64	14	5.8
Philips Guardian Body	12	5
Other scanners	16	6.7

^1^ Other lung cancer histology: NSCLC poorly differentiated lung carcinoma (4), NSCLC adenosquamous lung carcinoma (3), NSCLC neuroendocrine tumor (3), NSCLC mucoepidermoid carcinoma (2), NSCLC pleomorphic (1), NSCLC sarcomatoid carcinoma (1). Other PET/CT scanners: Siemens Biograph 128 (2), Siemens 1094 (6), Siemens SOMATOM (1), Philips Medical Systems GEMINI TF Big Bore (2), Philips Medical Systems Guardian Body (12), Philips Vereos (3), Philips Medical Systems GEMINI TF TO F16, (3), GE MEDICAL SYSTEMS Discovery 600 (1), CPS 1024 (1). BMI = body mass index, PET/CT = positron emission tomography/computed tomography, NSCLC = non-small cell lung cancer, SCLC = small cell lung cancer, COPD = chronic obstructive pulmonary disease.

**Table 2:** Influence of patient characteristics on metabolic activity of lung tissue. Spearman’s correlation analysis between standardized uptake values and clinical parameters. Adjusted alpha level after Bonferroni correction: *p*-value < 0.0025.

Variable	Sex	Age	BMI	Smoking	PY	COPD	Stage	OP	Rad	PE	PCE	DM	CHD	Hb
SUV_MAX_														
whole lung			**+**		**-**	**-**								
upper lung			**+**		**-**	**-**								
lower lung			**+**		**-**	**-**								
TFL			**+**			**-**								
SUV_MEAN_														
whole lung			**+**		**-**	**-**								
upper lung			**+**	**-**	**-**	**-**								
lower lung			**+**		**-**	**-**								
TFL			**+**			**-**								
SUV_95_														
whole lung			**+**		**-**	**-**								
upper lung			**+**		**-**	**-**								
lower lung			**+**		**-**	**-**								
TFL			**+**			**-**								
SUV_MEAN_ lung/liver														
whole lung			**+**		**-**	**-**								
upper lung			**+**	**-**	**-**	**-**								
lower lung			**+**			**-**								
TFL			**+**			**-**								
SUV_MEAN_ lung/blood pool														
whole lung			**+**		**-**	**-**								
upper lung			**+**	**-**	**-**	**-**								
lower lung			**+**			**-**								
TFL			**+**			**-**								
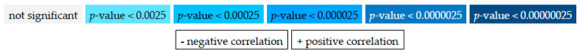														

Sex = male, BMI = body mass index, PY = pack years, COPD = chronic obstructive pulmonary disease, stage = tumor stage, OP = operation, Rad = thorax radiation, PE = pleural effusion, PCE = pericardial effusion, DM = diabetes mellitus type II, CHD = coronary heart disease, Hb = blood hemoglobin level, SUV = standardized uptake value, TFL = two left or right spheres depending on tumor side. For additional details, please refer to the Supplementary Materials (S1).

## Data Availability

Research data and code can be found and downloaded at https://doi.org/10.5282/ubm/data.454 (accessed on 15 May 2024).

## Supplementary Materials

The following supporting information can be downloaded at: https://www.mdpi.com/article/10.3390/diagnostics14111139/s1; Excel File S1: Data Used in Table 2 (Separate Sheets for *p*-values, Estimates, and Correlations); Excel File S2: Data Used in Table A2 (Separate Sheets for *p*-values, Estimates, and Correlations); Figure S3: Forest plot of patients scanned with GE Discovery 690.

## Appendix A

**Table A1:** Comparing SUV measurements regarding PET-CT scanner.

SUV Measurements	*p*
SUV_MAX_	
whole lung	0.03 *
upper lung	0.1603
lower lung	0.0114 *
TFL	0.0069 **
SUV_MEAN_	
whole lung	0.3562
upper lung	0.2033
lower lung	0.2462
TFL	0.1932
SUV_95_	
whole lung	0.3664
upper lung	0.5909
lower lung	0.104
TFL	0.0745
SUV_MEAN_ lung/liver	
whole lung	0.2663
upper lung	0.0534
lower lung	0.1863
TFL	0.0724
SUV_MEAN_ lung/blood pool	
whole lung	0.0139 *
upper lung	0.0044 **
lower lung	0.0166 *
TFL	0.0062 **

SUV = standardized uptake value, TFL = two left or right spheres depending on tumor side, adjusted alpha after Bonferroni correction: *p*-value < 0.0025, * = *p*-value < 0.05, ** = *p*-value < 0.01.

## Appendix B

**Table A2:** Influence of patient characteristics on metabolic activity of lung tissue for patients scanned with GE Discovery 690 (*n* = 137).

Variable	Sex	Age	BMI	Smoking	PY	COPD	Stage	OP	Rad	PE	PCE	DM	CHD	Hb
SUV_MAX_														
whole lung			+			-								
upper lung			+											
lower lung			+											
TFL			+											
SUV_MEAN_														
whole lung			+			-								
upper lung			+		-	-								
lower lung			+			-								
TFL			+			-								
SUV_95_														
whole lung			+			-								
upper lung			+		-	-								
lower lung			+			-								
TFL			+											
SUV_MEAN_ lung/liver														
whole lung			+			-								
upper lung			+			-								
lower lung			+			-								
TFL			+											
SUV_MEAN_ lung/blood pool														
whole lung			+			-								
upper lung			+			-								
lower lung			+											
TFL			+											

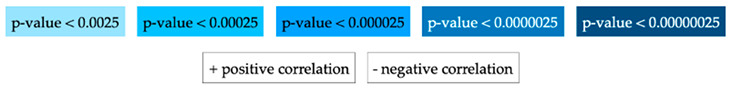

Sex = male, BMI = body mass index, PY = pack years, COPD = chronic obstructive pulmonary disease, stage = tumor stage, OP = operation, Rad = thorax radiation, PE = pleural effusion, PCE = pericardial effusion, DM = diabetes mellitus type II, CHD = coronary heart disease, Hb = blood hemoglobin level, SUV = standardized uptake value, TFL = two left or right spheres depending on tumor side, adjusted alpha after Bonferroni correction: *p*-value < 0.0025. For additional details, please refer to the Supplementary Materials (S2).
