# Supplementary figures and images for: The Association between the Body Mass Index, Chronic Obstructive Pulmonary Disease and SUV of the Non-Tumorous Lung in the Pretreatment [18F]FDG-PET/CT of Patients with Lung Cancer
Source: Diagnostics (Basel). 2024 May 30;14(11):1139. doi: 10.3390/diagnostics14111139 (PMC11171792; doi:10.3390/diagnostics14111139)

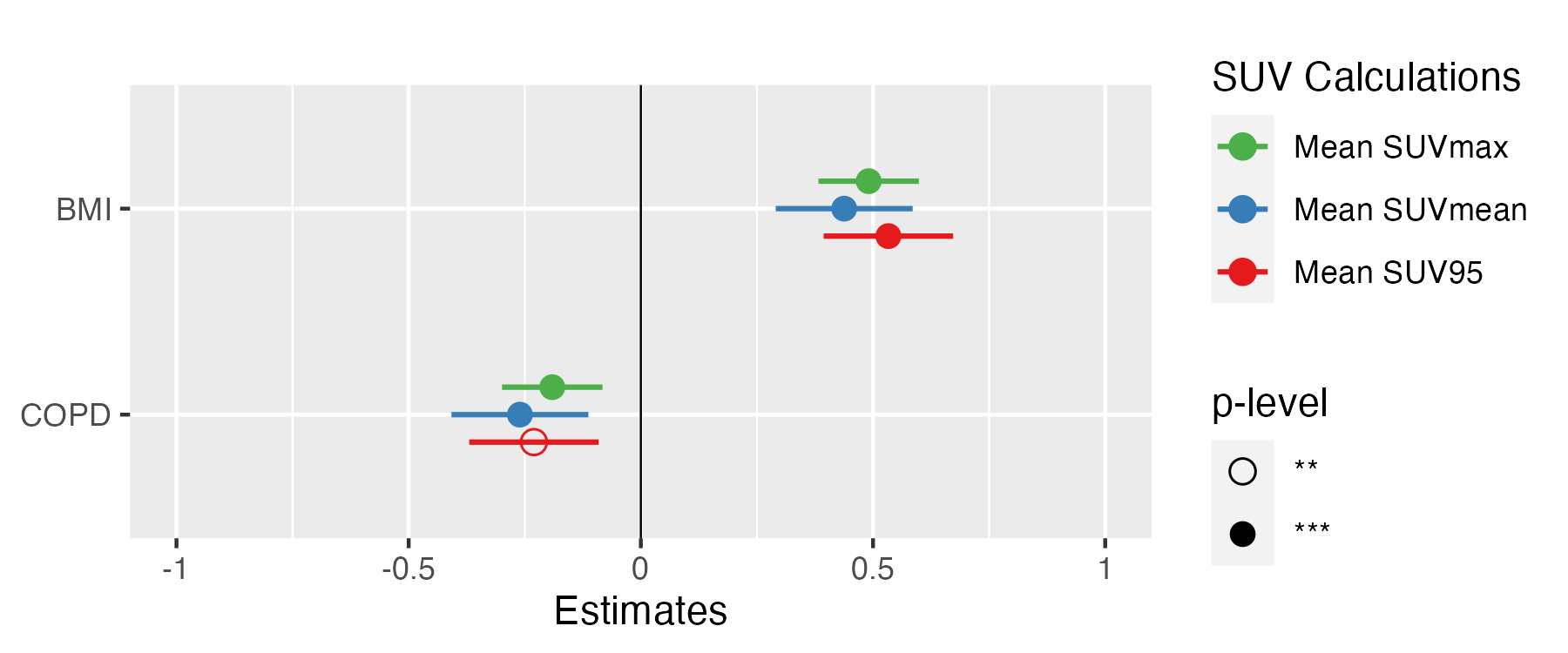

Supplement: Supplementary file 1 [file diagnostics-14-01139-s001.zip › Supplementary Files/s3.png]
